# Reporting oncoimaging in the appropriate oncology setting

**DOI:** 10.1186/1470-7330-15-S1-P32

**Published:** 2015-10-02

**Authors:** S De Luca, C Carrera, E Casalini Vañek, L Tolkachier, L Alarcon, E Eyheremendy

**Affiliations:** 1 Hospital Aleman, Buenos Aires, Argentina

## Learning objectives

Demonstrate the importance of multidisciplinary interrelationship between the oncologist and the radiologist for correct imaging interpretation.

Illustrate in a pictorial essay scenario, cases in which, considering clinical condition, laboratory values, complementary examinations and evolution of lesions on imaging exams are essential for an adequate presumptive diagnosis.

## Content organisation

The cases we will present include:

Young male patient with malignant germ cell tumour, treated with bleomycin. With pulmonary small nodules in a control MDCT suspected of metastasis in the setting of negative humoral markers and surgical biopsy confirmed drug hypersensitivity.

Male patient with mixed germ cell tumour in control MDCT which derived to PET CT demonstrated new hypermetabolic lymphadenopathy of mediastinum without elevated humoral markers and biopsy by mediastinoscopy confirmed sarcoidosis.

Patient with colorectal adenocarcinoma with lung, liver and adrenal metastasis in the context of onset of dyspnea. According to RECIST criteria represented a pulmonary stable disease but one of the nodules produced greater extrinsic compression of the nearest main bronchus so we considered real progressive disease clarifying that we did not take into account the classical criteria.

A woman with locally advanced colorectal adenocarcinoma with rapidly progressive multinodular pleuropulmonar, brain commitment and fever. We suspected infectious origin and resulted nocardia concomitant infection.

## Conclusion

We emphasise the importance of adequate clinical information and multidisciplinary interrelationship for the correct imaging interpretation in oncological patients.

